# Structural changes in the side branches and the circle of Willis following the use of flow-diverting stents

**DOI:** 10.55730/1300-0144.5397

**Published:** 2022-03-13

**Authors:** Hasan Bilen ONAN, Umur Anıl PEHLİVAN, Sinan SÖZÜTOK, Sevgül KÖSE, Erol AKGÜL

**Affiliations:** 1 Department of Radiology, Medicine Faculty, Çukurova University, Adana, Turkey; 2 Department of Radiology, Medicine Faculty, İstanbul Medipol University, İstanbul, Turkey

**Keywords:** Flow-diverting stents, circle of Willis, intracranial aneurysm

## Abstract

**Background/aim:**

This study aimed to evaluate the diameter and flow changes in the circle of Willis and side branches following the use of FDSs extending from the middle cerebral artery (MCA) to the internal carotid artery (ICA) for the treatment of aneurysms in the terminal segment of ICA, and the clinical results.

**Material and method:**

This study was conducted in a single center between January 2012 and April 2018 in patients with the anterior choroidal artery (AChoA), the posterior communicating artery (PComA), and the ICA terminal segment aneurysms treated with the FDSs. The changes in aneurysm size, arterial structures covered by the FDSs, and changes in the diameter and flow in arteries forming the circle of Willis were retrospectively analyzed.

**Results:**

Fourteen patients with a total of 25 aneurysms treated with FDSs extending from MCA to ICA were evaluated. The mean aneurysm fundus size was 5.14 mm (range 1.5–22 mm). Before treatment, the anterior communicating artery (AComA) was patent in all patients. Implanted FDSs covered the anterior cerebral artery (ACA) and AChoA in all patients (100%), nonhypoplasic PComA in two patients (14.28%), and the ophthalmic artery in nine (64.3%). The mean follow-up time was 36.78 ± 22.44 months. In follow-up, there was a decrease in the mean ipsilateral ACA A1 segment diameter from 1.99 ± 0.58 cm to 1.81 ± 0.31 cm (p = 0.01). The mean contralateral A1 segment diameter increased from 1.66 ± 0.48 cm to 1.93 ± 0.42 cm (p = 0.004). All aneurysms were totally occluded.

**Conclusion:**

If the AComA is patent, ipsilateral anterior circulation can be compensated through modifications in the contralateral ACA A1 segment in patients with ICA terminal segment aneurysms treated with FDSs extended from MCA to ICA and covering ACA. Although covering the anterior choroidal and lenticulostriate arteries by FDSs, ischemic complications may not occur frequently. Thus, this effective therapy can be applied more safely.

## 1. Introduction

Flow diverting stents (FDSs) alone or in combination with coil embolization are very important development for the treatment of technically challenging, wide-neck, and previously treated residual/recurrent aneurysms [1–9]. Most studies on FDSs have focused on whether the aneurysm can be successfully occluded. However, in recent years, one of the topics that have been increasingly emphasized in research is ischemic complications that may develop due to the side branches and perforating arteries covered by FDSs [9–11]. Ischemic complications that may arise with the use of FDSs, which have a smaller pore structure than conventional stents, especially in cases where they cover perforating, and major branches cause concerns. Animal experiments conducted to clarify this issue have used flow dynamics and advanced diagnostic methods and demonstrated that the covered branches could undergo modification when compensated by collateral arteries. There would be no significant diameter difference when collateral arteries are not functional [12–14].

This study aimed to investigate the diameter and flow changes in the arteries included in the circle of Willis following the use of FDSs extending from the middle cerebral artery (MCA) to the internal carotid artery (ICA) for the treatment of aneurysms in the ICA terminal segment and to evaluate the presence of clinical and radiological ischemic findings.

## 2. Material and methods

### 2.1. Study design and patient selection

This single-center, retrospective study was conducted between January 2012 and April 2018. Before the procedure, informed consent was obtained from the patients, and approval was received from the local ethics committee. Fourteen patients with 25 aneurysms of the ICA terminal segment, AChoA, and PComA, in which ACA was treated by covering with FDS, were included in the study. The inclusion criteria were having recurrent/residual aneurysms detected during the follow-up of endovascular or surgical treatment and/or having aneurysms considered to have a high risk of complications according to the assessment undertaken due to clinical and demographic and undergoing the endovascular procedure in which ACA was covered with the FDS. Arterial diameters and changes were calculated on 2D images. All cases were treated electively.

### 2.2. Medication

In all patients, 100 mg acetylsalicylic acid (ASA; Aspirin; Bayer Healthcare, Germany) and 75 mg clopidogrel (Plavix; Bristol-Myers Squibb/Sanofi Pharmaceuticals, NY, USA) were started daily at least seven days before treatment. The sensitivity of ASA and clopidogrel were evaluated with VerifyNow (Accumetrics, San Diego, CA, USA) one or two days before the treatment. According to the sensitivity of the antiplatelets, if Aspirin sensitivity was under the threshold, the dose was increased to 300 mg a day, and if clopidogrel sensitivity was under 30%, clopidogrel was replaced with ticlopidine 250 twice daily (Ticlocard; Kocak Farma, Istanbul, Turkey), ticagrelor 90 mg twice daily or prasugrel 10 mg daily was initiated in those without contraindications. Dual antiplatelet treatment was applied for at least six months. Subsequently, monotherapy was continued indefinitely, preferably with ASA.

### 2.3. Technique

All treatments were performed under general anesthesia. All procedures were performed by entering through the right femoral artery. A long 6F introducer, a five or 6F distal access guiding catheter, a 0.027-inch microcatheter, and a 0.014-inch micro-guidewire were used in accordance with the anatomy of ICA and the stent to be used. Six different stents were used in the treatment: Pipeline flex (Medtronic Covidien AG, Paris, France) (n = 2), Silk (Balt, Montmorency, France) (n = 3), Derivo (Acandis) (n = 5), Surpass (Stryker Neuroendovascular, Kalamazoo, MI, USA) (n = 2), two Pipeline Shield (Medtronic Covidien AG, Paris, France), and Derivo2 (Acandis, Pforzheim, Germany) (n = 1). Three Silk (Balt) stents were used in one patient, and two Surpass (Stryker) stents were used in another patient in the same session, while a single FDS was used in the remaining 12 patients.

3D rotational angiography and cone-beam CT (Artis zee biplane; Siemens Healthineers, Erlangen, Germany) were used to select the stent size. If the distance between the aneurysm neck and the ICA bifurcation was 5mm or less, the stent has been deployed starting from the MCA M1 segment.

The aneurysm neck was covered with an FDS extending to least five mm from the proximal and distal region of the aneurysm neck. The shortest stent was chosen not to cover many more arteries as far as possible.

At the beginning of the procedure, a bolus dose of 5000 IU IV heparin was administered. Then, an additional IV 1000 IU heparin was administered at each subsequent hour to ensure the activated clotting time was two to three times the baseline value. The patients were followed up clinically in the intensive care unit for 24 h after the procedure. During this period, heparin infusion was started at varying doses (500–750 IU/h) according to the patients’ weight, and antiplatelet treatments were continued.

### 2.4. Follow-up and statistical analysis

The first follow-up of all patients was undertaken by digital subtraction angiography (DSA) in the third or sixth month. Then, annual DSA follow-ups were conducted to evaluate aneurysm occlusion and stent patency. MRI was performed urgently in cases of symptoms suggesting stent thrombosis after treatment. Although there were no symptoms, all patients were evaluated with diffusion-weighted MRI for occult ischemia postprocedure first day. In one patient that did not want to have a DSA after the sixth-month examination due to the total occlusion, complete stent patency, and symptom relief, the maximum follow-up period was limited to six months when performing the statistical analysis. However, the follow-up of this patient was continued with computed tomography and magnetic resonance angiography.

During all follow-ups, the covered arteries and the flow in the circle of Willis were evaluated on 2D images. Additionally, the diameter of the ipsilateral posterior cerebral artery (PCA) P1 segment was recorded. The occlusion of aneurysms was assessed based on the Raymond-Roy classification.

IBM SPSS Statistics version 20.0 package program was used for the statistical analysis of the data. Categorical measurements were summarized as numbers and percentages, and numerical measurements as mean and standard deviation (minimum-maximum where necessary). The Wilcoxon signed-rank test compared two dependent numerical measurements that did not show normal distribution. The statistical significance level was taken as 0.05 in all tests.

## 3. Results

**Figure 1:**
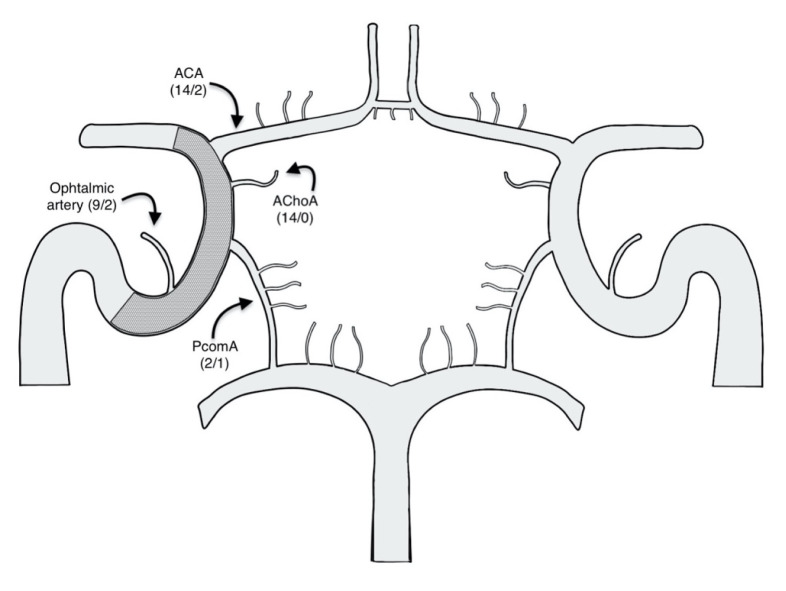
Schematic drawing of the relationship between the arterial structures in the circle of Willis and flow-diverting stents. (*/**) *: Number of patients defined to have patent arteries before the procedure. **: Number of patients that developed occlusion after the procedure. ACA: Anterior cerebral artery. AChoA: Anterior choroidal artery. PcomA: Posterior communicating artery.

A total of 25 aneurysms were identified: 18 in the ICA terminal segment (72%), five in the AChoA orifice (20%), and two in the PComA orifice (8%) (Table 1). The mean size of the aneurysm fundus was measured as approximately 5.14 mm (1.5–22 mm). The sample consisted of 11 female (78.6%) patients and three male (21.4%). In the treatment of aneurysms in all patients, at least one FDS was used. In six (24%) of the 25 aneurysms, a combination of an FDS and coil embolization was utilized. AComA was patent in all cases, while ipsilateral PComA patency was detected in two patients. In the remaining 12 patients, the ipsilateral PComA was hypoplasic. After treatment, the FDSs were seen to have covered AChoA and ACA in 14 patients, PComA in two (14.28%), and the ophthalmic artery in nine (64.3%) (Table 2, Figure 1).

**Figure 2:**
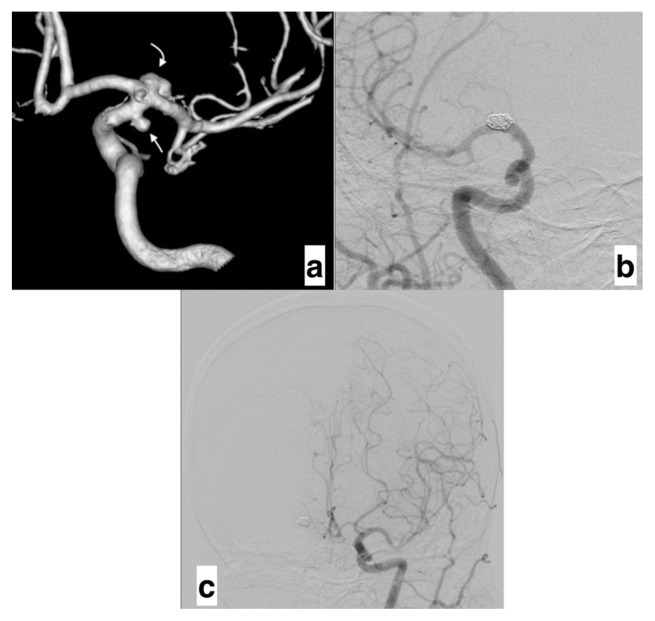
a) 3D images show the existing aneurysms in the right ICA terminal (curved arrow) and AChoA orifice (straight arrow). b) Postprocedural images reveal the occluded ipsilateral ACA. c) Anterior circulation is compensated by the increase in the diameter of the contralateral ACA. (ICA: Internal carotid artery, ACA: Anterior cerebral artery, AChoA: Anterior choroidal artery).

The mean follow-up was 36.78 ± 22.44 months (range, 6–94 months). During the follow-up, it was determined that there was a decrease in the mean diameter of the ipsilateral ACA A1 segment from 1.99 ± 0.58 cm to 1.81 ± 0.31 cm (p = 0.01). In contrast, the mean diameter of the contralateral A1 segment increased from 1.66 ± 0.48 cm to 1.93 ± 0.42 cm (p = 0.004). For the ipsilateral ACA, occlusion was seen in two patients (14.28%) (Figure 2 a-c), while the remaining 12 patients (85.71%) presented with a reduced diameter and in the flow on DSA. In contrast, on the contralateral ACA A1 segment, there was an increase in diameter and flow in 13 patients (92.85%). At the same time, no significant change was noted in the diameter or DSA flow in the remaining patient compared to the pretreatment values.

In nine patients (64.28%), the FDS covered the ophthalmic artery, and the mean diameter of the covered arteries decreased from 0.66 ± 0.19 cm to 0.51 ± 0.33 cm (p = 0.068). Ophthalmic artery occlusion developed in two patients; however, no visual symptoms developed in either case. Before the procedure, two of 14 patients had ipsilateral PComA patency. In both cases, the artery was covered with an FDS, and one was occluded after the procedure. Both PComA cases were also nonfetal. The mean diameter of the ipsilateral PCA P1 segment was observed to have increased from 1.81 ± 0.31 cm to 1.86 ± 0.29 cm (p = 0.899).

**Figure 3:**
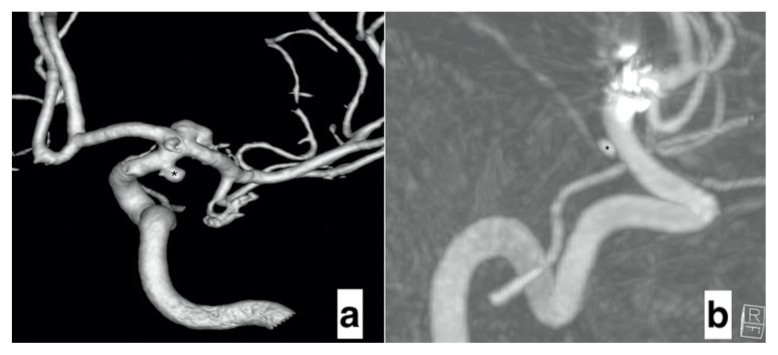
a) Preprocedural images of the aneurysms in the ICA terminal segment (*) and AChoA orifice using flow diverting stents and additionally a coil in the terminal segment. b) Remodeling images in which the aneurysm in the AChoA orifice (*) is significantly reduced in size but remains patent due to the perforating arteries originating from the neck (ICA: Internal carotid artery. AChoA: Anterior choroidal artery).

Fourteen ACA and AChoA, nine ophthalmic arteries, and two PComA were covered by FDSs (Table 2, Figure 1). AChoA was covered with a flow diverting stent in all patients, but none developed occlusion in this artery. In one patient, AChoA originated from the aneurysm neck (Figure 3a). But there was no residual filling in the aneurysm, although there was no decrease in calibration due to remodeling in AChoA compared to the preprocedural evaluation (Figure 3b). On the DSA that was received immediately after the procedure, there was no change in the flow form or diameter of the covered arteries, except one. The details of this case that showed changes were given in the periprocedural complication section. Additionally, in one more patient, proximal stenosis was detected at the 24th-month follow-up, and this complication is discussed in the late complication section.

In the 24th-hour control examination of the patients, no finding suggesting early-stage clinical ischemia was detected. Diffusion MRI was used to rule out silent ischemia, and very small foci of acute infarction were found in six patients (42.85%) without any clinical findings. It was determined that among the cases presenting silent ischemia, a single FDS had been used in five (83.3%) and two FDSs in two (16.7%). Silent ischemia was not detected in a case in which three FDSs had been implanted. In all patients with silent ischemia, the diffusion MRI findings were revealed diffusion restriction due to distal thromboembolism, not secondary to covered perforating arteries. Of 14 cases, 13 did not present any clinical sign of ischemia during the follow-up period (range, 12–94 months). The aneurysms were totally occluded in all cases. The related findings are summarized in Table 3.

### 3.1. Periprocedural complications

**Figure 4:**
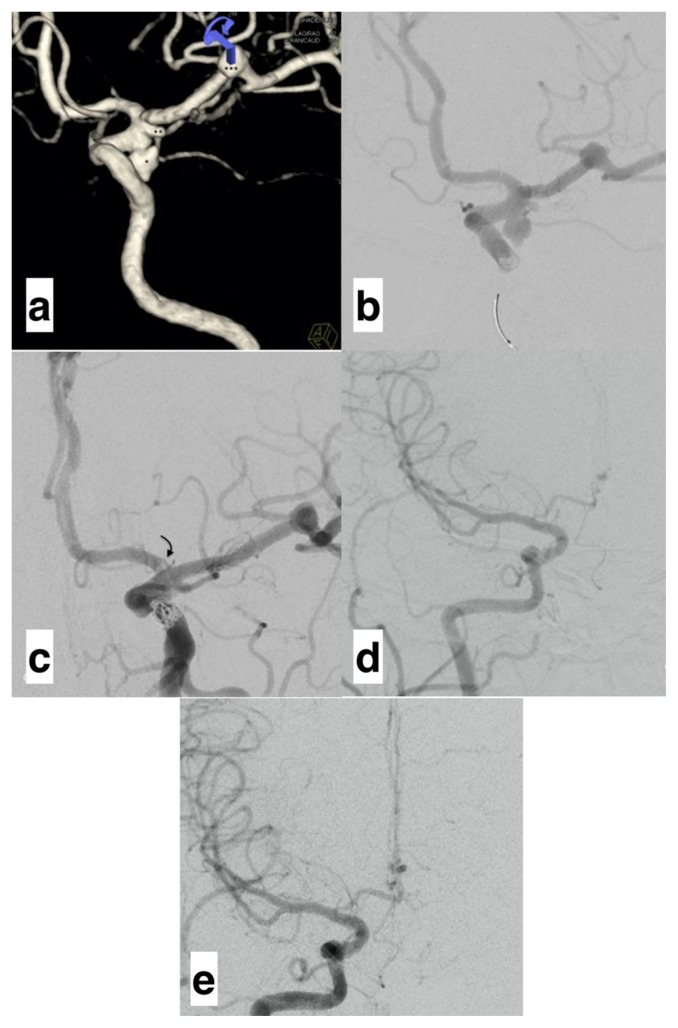
a,b) 3D and DSA images of the patient with two aneurysms in the ICA terminal (*,**) and one aneurysm in MCA bifurcation (***). c) Image obtained at the end of the procedure revealing a thrombus (curved arrow) in the ipsilateral ACA A1 segment, which does not block the flow. d) DSA obtained with contralateral carotid injection before the procedure. e) DSA obtained with contralateral carotid injection after the procedure, revealing increase in calibration after rapid modification in the ACA A1 segment (ICA: Internal carotid artery. ACA: Anterior cerebral artery. MCA: Middle cerebral artery).

No periprocedural major complication, such as rupture, thromboembolism, subarachnoid hemorrhage, or acute occlusion of covered arteries, was seen, except one. Patient 14 (Table 3) had two aneurysms in the ICA terminal segment and an MCA bifurcation aneurysm (Figure 4a, 4b). Before the procedure, the A1 segment of the contralateral ACA was hypoplasic (0.78 mm). The proximal one was partially filled with three coils for the ICA aneurysms. Then a 3.5 x 14 mm Pipeline Shield device was implanted, extending from MCA to ICA and covering both aneurysm necks. The MCA bifurcation aneurysm was also treated with an FDS. In the images taken after the procedure, a partial thrombus was detected in the ipsilateral ACA A1 segment, the flow of which was not completely blocked (Figure 4c). The thrombus persisted despite administering a total of 1 mg IV tirofiban. After contralateral femoral artery access, the contralateral carotid injection was applied, and images were obtained. The ACA A1 segment rapidly adapted to compensate for this situation, increasing its diameter from 0.78 to 1.1 mm (Figure 4d, 4e). It was determined that there was no problem in the blood supply to the ipsilateral ACA distal segment after the contralateral carotid injection; thus, the procedure was terminated. No complication, including occult ischemia, was detected during the follow-up.

### 3.2. Long term complications

There was no long-term complication in 13 patients (92.85%). In the single remaining case (7.14%), the 24^th^-month-follow-up DSA showed 75% narrowing in the proximal part of the FDS, which led to recurrent symptoms. Therefore, a Wingspan stent (Stryker, Kalamazoo- MI) was implanted. The follow-up examinations performed after the second procedure revealed no sign of in-stent thrombus, intimal hyperplasia, or clinical symptoms.

## 4. Discussion

Darsaut et al. conducted preclinical studies in 2012 and 2014 to evaluate FDSs in 21 and 17 experimental animals, respectively. In both studies, stents with a different number of wires were used in three variants of an aneurysm model (straight sidewall, curved sidewall, and end-wall bifurcation), and the FDSs were reported to be effective in the treatment of straight sidewall aneurysms but not incurved sidewall and end-wall bifurcation aneurysms [15,16]. However, we consider that the data from these studies could mislead readers with radical results obtained over a short period of only 12 weeks. Contrary to the implications of these preclinical studies, the efficacy and safety of FDSs on aneurysm occlusion have been proven by meta-analyses and case series in the literature [4–7,17,18].

Recent preclinical studies have attempted to explain the diameter and flow modifications in the arteries covered with FDSs. Iosif et al. conducted three studies on this subject. They measured the changes in the irrigation areas of the arteries covered with FDSs, as well as the alteration in the flow of these arteries using computational fluid dynamics. In addition to an optical coherence tomography (OCT) examination before euthanasia, the authors also performed an electron microscopic evaluation by dissecting the covered arteries after euthanasia [12–14]. In these studies, it was described that the patency of the arteries covered with FDS depended on the presence of an alternative collateral network in the irrigation area of the covered artery. There was also clinical research conducted without evaluation of electron microscopy and OCT, similarly indicating that symptomatic modifications in the covered arteries after treatment with FDS were based on the extent and type of collateral supply [19].

In light of large case series and meta-analyses, treatment of intracranial aneurysms with FDSs is a highly effective method in the occlusion of aneurysms [4–7,17,18]. However, there are still concerns arising from their denser metallic structure, higher surface coverage, and lower porosity than conventional stents used in intracranial aneurysm treatment [1], leading to complications in the irrigation areas of the arteries when applied. A case report was also available in the literature to justify experts’ concerns concerning this issue [20]. Due to all these concerns, new literature data were obtained in relation to the use of FDSs in cases that were technically difficult or almost impossible to treat with current treatment modalities. Until the last 5 years, studies on FDSs mainly focused on whether aneurysms were occluded. But some meta-analyses [4–7,9,10,18,21] and case series [8,11,19,22–24] that retrospectively compile data from defined the complications of this treatment modality and type of clinical problems it might cause. A meta-analysis including IntrePED, PUFS, and ASPIRe studies determined the major ipsilateral ischemic stroke rate as 3.7%, major ipsilateral intracranial bleeding as 2.0%, major neurological morbidity rate as 5.7%, mortality rate as 3.3%, and the combined rate of major neurological morbidity and mortality as 7.1% [9]. In the IntrePED study, it is found that acute ischemic stroke was associated with male sex, hypertension, treatment of fusiform and/or giant aneurysms, and use of multiple FDSs in the univariate analysis and only fusiform treatment in the multivariate analysis [10]. In our series, only one patient (Patient 4) experienced late recurrent ischemic symptoms, which reminded us of the importance of fusiform aneurysms described by Brinjikji et al. [10] in ischemic stroke (Table 3). The authors also stated that more catastrophic complications might occur in posterior circulation aneurysms compared to anterior circulation.

Studies have also investigated the specific patency of individual arteries covered. To our knowledge, although there are several studies on postprocedural modifications of the ophthalmic artery [25], PComA [26], and AChoA [27,28], there are two studies in the literature presenting data on all distal branches of the ICA [11, 29]. Recently, a newer study was documented about outcomes of ACA covered by FDSs [30]. Based on all these publications, the number of studies published in the literature is insufficient to evaluate the diameter and flow modifications in the anterior circulation and clinical follow-up after using FDSs to cover ACA. In a case series of 82 patients published by Rangel-Castilla et al. [29], ACA was covered by an FDS only in two cases. Both presented with asymptomatic occlusion, which was found during the follow-up. In the other case series, including 140 patients and 147 aneurysms reported by Bhogal et al., 14 patients had ACA covered by FDS. Two of these patients developed occlusion, and six had decreased flow [11]. A wider case series included 42 patients who had ACA covered by FDS. ACA narrowed or occluded in 35 of 42 patients [30]. However, the authors reported no symptoms during the follow-up. They noted that the distal ACA A2 segment in the patients with occlusion or narrowing was fed by AComA in contralateral circulation [11,29, 30]. Our study found decreased ipsilateral diameter and flow in 12 of 14 patients, and occlusion was present in two. DSA performed by contralateral carotid injection revealed that in the patients with occlusion, the ipsilateral ACA A1 and A2 segments were filled with AComA. The contralateral ACA A1 diameters of these patients increased, and it was also noted that the flow was visually increased in DSA images. Neurological deficits were not detected in any of these patients during the follow-up. An ipsilateral thrombus developed only in one case, described in the section on periprocedural complications. This patient could not be evaluated by a physical examination because she was under general anesthesia. The synchronous MCA bifurcation aneurysm of this patient was also treated in the same session. First, the ICA terminal segment aneurysms were treated with an FDS, and then the MCA aneurysm was treated through the same access route. We consider that this complication occurred because of the prolonged catheterization time, leading to increased thrombogenicity at the implantation site. A thrombus in the ipsilateral ACA in this patient did not completely cut off the flow but significantly decreased it. Upon observing no thrombus resolution despite 1 mg of IV tirofiban administration, DSA was undertaken with a contralateral carotid injection to evaluate whether anterior circulation was affected. This procedure showed that ipsilateral anterior circulation was not affected due to the flow compensation.

AChoA was covered by an FDS in all 14 patients, but no occlusion was detected in any of our cases, consistent with previous reports [11,26–29]. Only in one case, in which AChoA originated from the aneurysm neck, there was a decrease in the AChoA flow. Neither a decrease in diameter nor a decrease in flow was detected in any of our remaining patients. Furthermore, no patient presented with a neurological symptom, physical examination finding, or diffusion MRI sign suggesting ischemia in ganglionic and capsular localizations fed by AChoA. We consider that the acute ganglionic infarction described in a case report by Rooij and Sluzewski in 2010 was probably due to the use of two FDSs and technical complications related to the procedure [20]. Previous meta-analyses also associated such complications with the use of multiple FDSs in the univariate analysis, which can also explain the situation described by Rooij and Sluzewski [10].

The ophthalmic artery was covered by an FDS in nine of our cases, of which two were observed to have occlusion on the follow-up DSA. The diameter changes in this artery were statistically analyzed, and the reduction in diameter was not statistically significant. No ophthalmologic symptom was found in any of these patients, which can be explained by the presence of collateral support, probably provided by the external carotid artery. This finding was consistent with the data reported in the literature [11,25,26]. However, in their detailed ophthalmologic examination, Rouchaud et al. found a very high rate of optic atrophy and retinal artery thromboembolism, which were observed to progress subclinically [22]. The findings described by Rouchaud et al. could not be verified because a detailed ophthalmologic examination was not performed in our study.

Before the procedure, two of the 14 patients had ipsilateral PComA patency in this study. The PComA was covered with an FDS in both patients, and occlusion was observed in one patient after the procedure; however, there was no clinical sign of ischemia.

In our analyses, no statistically significant increase was found in the measurement of the mean diameter of the ipsilateral posterior cerebral artery P1 segment. However, we consider it inappropriate to generalize this finding since only two of our patients had nonfetal type PComA.

On the first day after the procedure, foci compatible with silent ischemia were found in diffusion-weighted MRI of six patients, but no additional medication was required. We evaluated the relationship of this parameter with the number of FDSs used. A single FDS was used in five of the six patients with diffusion restriction (83.3%) and two FDSs in the remaining patient (16.7%). Contrary to previous studies in the literature indicating a correlation between the number of FDS and the rate of clinically detected ischemia, the chi-square analysis of our data did not reveal a statistically significant correlation between silent ischemia and the number of FDS. However, due to the small sample size in our study and the fact that despite the absence of clinical symptoms in our patients, we investigated silent ischemia in more detail than in other studies, we consider that our statistical analysis results are not correlated with the literature. In the long-term, no neurological morbidity or mortality was detected during the clinical (12–94 months) or radiological (6–94 months) follow-up based on DSA in any of our patients.

### 4.1. Study limitations

The main limitations of this study were the number of cases and retrospective. Other limitations included the absence of an OCT examination, the gold standard for evaluating covered arteries in animal studies, the inability to analyze computational fluid dynamics [31], and the lack of a detailed ophthalmologic examination. Additionally, the DSA of the selective external carotid and vertebral arteries, which would allow collateral networks to be more clearly demonstrated, was not obtained for all patients.

## 5. Conclusion

Since the sample size is small, it is not possible to make generalizations based on our data; however, we observed structural changes in the circle of Willis, especially ACA, following the use of FDSs extending from MCA to ICA. If AComA is patent, anterior circulation can be compensated through the modifications in the contralateral ACA A1 segment. Thus, this effective treatment can be applied more safely.

## Figures and Tables

**Table 1:** Localizations of aneurysms.

Localization	Number
ICA terminal segment (C7)	18
AChoA orifice	5
PcomA orifice	2

ICA: Internal carotid artery. AChoA: Anterior choroidal artery. PcomA: Posterior communicating artery.

**Table 2:** Arteries covered with flow-diverting stents and their occlusion status in the last follow-up.

	Number of covered arteries	Number of occlusions
ACA	14	2
AChoA	14	0
PcomA	2	1
Ophthalmic artery	9	2

ACA: Anterior cerebral artery. AChoA: Anterior choroidal artery. PcomA: Posterior communicating artery.

**Table 3:** Diameter changes before and after flow-diverting stent implantation and evaluation of response to aneurysm treatment.

	Patient 1	Patient 2	Patient 3	Patient 4	Patient 5	Patient 6	Patient 7	Patient 8	Patient 9	Patient 10	Patient 11	Patient 12	Patient 13	Patient 14
ACA A1 diameter on the an. side before procedure (mm)	2.15	2.5	2.7	2.4	2.3	2.1	1.73	1.5	2.2	0.9	1.85	2.07	0.85	2.6
ACA A1 diameter on the an. side after procedure (mm)	1.06	0.9	0	0	1.4	0.5	1.1	0.46	1.24	0.7	0.92	1.04	0.72	1.07
Contrateral ACA A1 diameter before the procedure (mm)	1.74	0.8	1.4	2.2	2.1	1.6	1.3	2.12	1.6	1.6	1.7	2.06	2.26	0.78
Contrateral ACA A1 diameter after procedure (mm)	2	1.4	1.5	2.76	2	2.28	1.82	2.3	1.93	1.76	1.92	2.05	2.21	1.07
PCA P1 diameter on the an. side before procedure (mm)	2.07	2	1.8	1.7	1.8	1.9	1.35	1.7	1.45	2.18	2.39	2.05	1.63	1.34
PCA P1 diameter on the an. side after procedure (mm)	2.07	2	1.8	1.8	1.8	2.21	1.64	1.7	1.45	2.15	2.25	2	1.5	1.33
OA diameter before procedure (mm)	0.9	0.6	0.5	0.5	0.6	0.4	0.6	0.68	0.6	0.85	0.82	1.04	0.68	1.11
OA diameter after procedure (mm)	0.9	0.9	0.5	0.6	0.6	0.4	0	0.6	0.6	0.83	0.9	0.95	0	1.11
PcomA diameter before procedure (mm)	0	0	0.8	0	0	0	0	0	0	0	0	0	0	0.78
PcomA diameter after procedure (mm)	0	0	0	0	0	0	0	0	0	0	0	0	0	0.8
Raymond-Roy classification	TO	TO	TO	TO	TO	TO	TO	TO	TO	TO	TO	TO	TO	TO
Branches covered by FDS*	1,2,3,4	1,4	1,2,3,4	1,2,4	1,2,3,4	1,2,3,4	1,2,3,4	1,2,3,4	1,2,3,4	1,2,4	1,2,4	1,2,3,4	1,2,3,4	1,2,4
Number of an.	3	1	3	1	2	1	2	1	3	1	1	3	1	2
Presence of silent infarction on MRI on the first day after procedure	−	−	−	+	−	−	+	+	−	+	−	−	+	+
Type of FDS**	1	2	3	4	3	3	4	3	3	6	2	2	5	5
Number of FDS/FDSs use	1	1	1	1	1	1	2	1	1	1	1	3	1	1

* 1: ACA (Anterior cerebral artery) 2: PcomA (Posterior communicating artery) 3: OA (Ophthalmic artery) 4: AChoA (AChoroidal artery)

** Pipeline flex 2: Silk 3: Derivo 4: Surpass 5: Pipeline Shield 6: DerivoII

An: Aneurysm. FDS: Flow diverting stent. PCA: Posterior cerebral artery. TO: Totally occluded.
